# Anisotropic non-rigid Iterative Closest Point Algorithm for respiratory motion abdominal surface matching

**DOI:** 10.1186/s12938-019-0643-4

**Published:** 2019-03-18

**Authors:** Dominik Spinczyk, Mateusz Bas

**Affiliations:** Biomedical Engineering, Silesian University of Technology, 40 Roosevelta, 41-800 Zabrze, Poland

**Keywords:** Non-rigid Iterative Closest Point, Anisotropic Iterative Closest Point, Time of Flight noise model

## Abstract

Surface registration is a one of the crucial and actual problems of computer aided surgery. This paper presents the modification of the non-rigid Iterative Closest Point Algorithm which takes into account an anisotropic noise model and landmarks as guided correspondence at the transformation step in every iteration. The presented approach was validated on human abdominal briefing surface data from a time-of-flight camera. We took the median of the resulting measures and the outcome is presented: the median of means of surfaces distance was at the same level for both variants of the ICP algorithm and is comparable with the isotropic variant, the median of mean landmark position errors decreased by 0.93 units (over 20% improvement) and the median of percentage of single correspondences in target point cloud increased by 11.96%. The results showed that the introduction of the anisotropic model of noise for the ToF camera allows for the improvement the percentage of target cloud points which had only one correspondent over 10% impartment and additional weighting of markers also improves the measure of the quality of finding real correspondents over 20% improvement. In the examined dataset, where the average initial distance between the clouds of points in the inspiratory and expiration is equal to approx. 7.5 mm, a more than 10% improvement in the quality of the correspondence improves the accuracy of matching the surface within 1 mm which is a significant value in application of minimally invasive image guided interventions.

## Introduction

Nowadays there’s more and more research carried out in the field of precise and automatized acquisition of human organs as 3D models. Such models can be used to diagnose a pathological state of an organ or enhance therapeutically procedures. A common use of 3D models of human body organs is minimally invasive surgery. Certain cases of working with 3D models require registration of point clouds for achieving an outcome in form of transformation values. That is where registration algorithms play a major role. Next in the few paragraphs we introduce Iterative Closest Point algorithm, then the rigid and non-rigid variants of the ICP algorithm and their recently published applications are presented.

One of the common known registration algorithms is the ICP algorithm introduced by [1]. The original algorithm preconceives that the input point cloud can only be applied with an affine transformation, resulting in the point cloud being considered rigid. The selection stage of the algorithm takes every point of the set into consideration. The matching stage is based on least Euclidean distances between points. The calculation of transformation is accomplished using least squares minimization of the distances between corresponding point pairs.

Modifications of the *rigid* Iterative Closes Point lead to the creation of two pathways of algorithm development. One alternative was presented by [2] and the main assumption was that the cloud to be fitted is non-rigid, which means that it can no longer be only applied with affine transformations. The points may move independently, but are constrained by the stiffness term. A second modification was introduced by [3], in which the rigid transformation remained but point pairing methods and transformation calculations took anisotropic point localization error into account. In the next two paragraph we focuses on *non*-*rigid application and ICP non*-*rigid algorithm modifications.*

In this section we present other recently announced and available *non*-*rigid application* for surface registration algorithms. A non-rigid surface registration algorithm which uses feature points and deformation constraint was presented by [4]. The algorithm focuses on extracting a set of feature points between two point clouds using Intrinsic Shape Signature and Heat Kernel Signature for acquiring four key corresponding points. The deformation of the point clouds must be limited within isometric point of views which allows for measuring geodesic distances between points to establish stable correspondences. Article [5] describes a 3D non-rigid registration using a color enhanced Coherent Point Drift algorithm. This algorithm uses Gaussian Mixture Models and Expectation Maximization to calculate corresponding similarities between two point models and also takes color of the points into account. A Point-Based Non-Rigid Surface Registration algorithm was proposed in [6]. By constructing a surface model from training samples of X-CT images and normalizing the shape, size and location of structures in images, the algorithm is able to provide a probabilistic distribution of corresponding points. This allows to register a surface model to a surface of internal organ in a 3D image. Article [7] reviews the Thin Plate Spline Robust Point Matching algorithm. This uses Soft assign and Deterministic Annealing to transform discrete correspondence between two subsets points into continuous one and solves transformation problem with least-square optimization. Another approach for non-rigid surface registration was presented by [8]. In the article, a registration of planning 3D CT images and treatment 3D cone-beam CT images is described. Proposed algorithm uses landmarks acquired from Active Shape Model, Laplacian Surface Deformation and Laplacian Surface Optimization for local deformation and remeshing of the models and generating transformation by minimizing the quadratic energy.

As regard recently published *ICP non*-*rigid algorithm modifications* we also have found some propositions. First novel enhancement to Iterative Closest Point Algorithm is provided by [9]. The article describes Cluster Iterative Closest Point which adapts the classic scheme of the algorithm. In the beginning CICP estimates normals of each point in both source and target set and divides the sets into voxels. Then single point in each voxel is elected as a representative based on uniform spatial grouping of the voxels and normal directions. Matching is performed using k–d trees and calculating nearest correspondences. Transformation of clouds is computed with Gauss–Newton iterative least square algorithm. Another variant of the ICP is presented in article [10]. The proposed modification uses a two-way correspondence matching scheme, meaning that corresponding points are being searched from source to target point cloud and inversely, from target to source, rather than the one-way approach. Instead of minimizing a simple quadratic energy function, authors propose using Laplacian-based potential energy function. After the review of the recently published application and non-rigid ICP algorithm modifications, in the next paragraph we present the purpose of the work.

The aim of this article is to present a modification of non-rigid Iterative Closest Point Algorithm by introducing an anisotropic noise model for the Time of Flight camera. Our article extends/continues research carried out by [11]. We present the application of the developed non-rigid ICP modification for tracking and predicting abdominal organs displacement in patients.

## Materials and methods

In this section we present previous works that have been our inspiration during creation of our modifications of non-rigid ICP algorithm.

### Non-rigid ICP for deformable surface registration

The non-rigid ICP is a variant of the classical rigid ICP which was presented by [1]. The new version of algorithm was introduced by [2] as a new approach which, assumes that the source cloud is capable of deformation due to the stiffness term. Source cloud vertices v:

1 $$v_{i} = \left[ {xyz1} \right]$$

are arranged such that the first three values of the vector are the x, y, z coordinates of the point. The last value should be ‘one’. This is due to the characteristic of the 4N × 3 transformation matrix:

2 $$X_{n} = \left[ {\begin{array}{*{20}c} {r_{11} } & {r_{12} } & {r_{13} } \\ {r_{21} } & {r_{22} } & {r_{23} } \\ {r_{31} } & {r_{32} } & {r_{33} } \\ {t_{1} } & {t_{2} } & {t_{3} } \\ \end{array} } \right]$$

When multiplying the vertex vector and its transformation matrix the bottom row of the rotation matrix corresponds to the ‘one’ values in the vertices vectors and allows the translation of the point. The other values of the transformation matrix allow the rotation in x, y and z axes.

An iteration of the algorithm starts with a search for correspondences between points of both the source and target clouds. This stage is the same as in rigid ICP algorithm. Euclidean or normal-shooting approach is available to find closest point pairs. After finding corresponding pairs of points we can define the distance term that has been proposed by [2] as follows:

3 $$E_{d} \left( X \right) = \left\| {W\left( {DX - U} \right)} \right\|^{2}$$

where E_d_—distance function cost, W—N × N identity matrix, D—N × 4N source cloud points, U—N × 3 correspondent target cloud points, X—4N × 3 transformation matrix.

In Eq. (3) the matrix W can initially be set the identity matrix and matrix D is defined in following way:

4 $$D = \left[ {\begin{array}{*{20}c} {v_{1}^{T} } & a & a & a \\ a & {v_{2}^{T} } & a & a \\ a & a & \ddots & a \\ a & a & a & {v_{n}^{T} } \\ \end{array} } \right]$$

Next two additions to the global cost function were presented as an improvement to the classical approach.

One of them is the *landmark term* which is similar to the distance term, but applied to landmark vertices. Matrices D_L_ and U_L_ are composed in the same way as the ones in correspondents distance term, except these matrices are filled with only vertices provided as pairs of landmarks. The beta parameter has been initially set to ‘one’. The landmark term is as follows:

5 $$E_{l} \left( X \right) = \beta \left\| {D_{L} X - U_{L} } \right\|^{2}$$

where E_l_—landmark distance function cost, D_L_—source cloud landmarks, U_L_—correspondent target cloud landmarks, X—transformation matrix.

Second of them is the stiffness term, which sets the rigidness of the source cloud. It needs to be provided with a topology matrix M which describes either 4-connected or 8-connected neighborhood relations between adjacent points in a mesh. The stiffness term is defined in following way:

6 $$E_{s} \left( X \right) = \alpha \left\| {\left( {M \otimes G} \right)X} \right\|^{2}$$

where α—stiffness parameter, E_s_—stiffness function cost, M—node-arc incidence matrix, G—4 × 4 weighting matrix, X—4N × 3 transformation matrix.

The matrix M should be defined such that each column represents a point in source cloud and rows represent single connectivity relation between these points. The relation is directional. In the column representing the starting point, a value of ‘− 1’ was used and in the column of the destination point, a value of ‘1’ was used. The rigidness of the cloud depends directly on the Alpha parameter. The matrix **G** can initially be set as the identity matrix. The operation done on the M and G matrices is the Kronecker product.

Finally we create the global cost function which is composed of the three terms described above. The global cost function is presented in following way:

7 $$E\left( X \right) = \left\| {\left[ {\begin{array}{*{20}c} {\alpha \left( {M \otimes G} \right)} \\ {WD} \\ {\beta D_{L} } \\ \end{array} } \right]X - \left[ {\begin{array}{*{20}c} 0 \\ {WU} \\ {U_{L} } \\ \end{array} } \right]} \right\|^{2} = \left\| {AX - B} \right\|^{2}$$

The matrix equation defined above is a quadratic function with an unknown variable X. It is possible to minimize that function by setting its derivative to zero and solving the acquired linear equations system. The minimum of the global cost function, with X as input variable, is located at:

8 $$X = \left( {A^{T} A} \right)^{ - 1} A^{T} B$$

Resulting transformations are applied to the source cloud points and it becomes the new initial input cloud for the algorithm. After that, a new iteration begins. The algorithm is repeated until either maximal iteration count has been achieved or clouds distance measure reaches a preset precision.

### Anisotropic ICP

Another innovative approach in terms of enhancement of the rigid ICP algorithm was proposed by [3]. This version of the algorithm assumes that the input point clouds contain normally distributed, zero-mean anisotropic point localization error. Few methods for computation of the covariance matrix of the noise model have been proposed. In this part, the description is limited to the Time-of-Flight noise model which has been used in this modification of ICP algorithm.

The time-of-flight localization error model assumes that the camera which the model was acquired from generates a point localization noise. It is a common issue with cameras and it is greater in the axis that connects the point and the camera origin than in perpendicular axes. The noise strength is set using variances that can be established arbitrarily or in accordance with the camera specification. Variance values are placed in a variance matrix which next is used to calculate covariance matrices $\varSigma_{{x_{i} }}^{k}$, $\varSigma_{{y_{i} }}$ and cross-covariance matrix $\varSigma_{ij}^{k}$ in each iteration:

9 $$\varSigma_{{x_{i} }}^{0} = V_{{x_{i} }} S_{{x_{i} }}^{2} V'_{{x_{i} }}$$

10 $$\varSigma_{{y_{j} }} = V_{{y_{j} }} S_{{y_{j} }}^{2} V'_{{y_{j} }}$$

where $\varSigma_{{x_{i} }}$—covariance matrix for source cloud, $\varSigma_{{y_{i} }}$—covariance matrix for target cloud, S—diagonal standard deviation matrices for source and target cloud, V—matrices of principal axes of localization error (columns represent axes).

It is important to notice that presented values of the cross-covariance matrix and the covariance matrix of source cloud are only initial. These values change with each iteration and the covariance matrix needs to be recalculated based upon the rotation values and values of the covariance matrix achieved in the previous iteration:

11 $$\varSigma_{ij}^{k} = \varSigma_{{x_{i} }}^{k} + \varSigma_{{y_{j} }}$$

12 $$\varSigma_{{x_{i} }}^{k} = R^{k - 1} \varSigma_{{x_{i} }}^{k - 1} \left( {R^{k - 1} } \right)^{'}$$

where $\varSigma_{{x_{i} }}$—covariance matrix for source cloud, $\varSigma_{{y_{i} }}$—covariance matrix for target cloud, $\varSigma_{ij}^{k}$—cross-covariance matrix, R^k−1^—rotation matrix.

Cross-covariance matrix allows the algorithm to calculate the weighting matrix W.

It is then used to modify the correspondents matching stage of the classical ICP algorithm. Article [3] assumes closest Euclidean distances between source and target cloud points. The weighting matrix W is calculated as follows:

13 $$W_{ij}^{k - 1} = w\left( {\varSigma_{ij}^{k} } \right)^{{ - \left( {\frac{1}{2}} \right)}}$$

where W—weighting matrix, $\varSigma_{ij}^{k}$—cross-covariance matrix, w—normalization constant.

The modification of the Euclidean distances by application of the weighting matrix leads to a new set of distances used to find correspondent points in source and target clouds:

14 $$d_{new} \left( {x,y} \right) = \left\| {W_{xy} \left( {x - y} \right)} \right\|$$

where d_new_—modified Euclidean point distances, W—weighting matrix, x—source cloud points coordinates, y—target cloud points coordinates.

Another alternation of the ICP algorithm presented in [3] is related to the weighting of points at the stage of calculating transformation matrix but it will not be reviewed due to a different method of weighting the points of a mesh introduced in this article.

### Proposal of addition of point localization error model and modification of landmark weighting

As mentioned in the introduction, this article is a continuation for work presented by [11]. This approach extends the methods shown in the referenced article by taking the algorithms of both [2] and [3] and creating a method of registering point clouds that would allow the model to remain non-rigid but would also take the point localization error into consideration. The algorithm is based on non-rigid registration algorithm while altering a couple of details.

#### Time-of-flight point localization error model

First, the corresponding points pairs finding stage was changed. Instead of using the plain Euclidean distance norm presented by [2], we modified the norm as equation [15] states. To calculate the correspondents distance weighting matrix $W_{xy}$, a point localization error is needed in the form of standard deviations on principal axes. This information should be known to parametrize the algorithm. From the details provided by the specification of the TOF camera, the localization error variance in z axis was acquired. The axis which connects the origin of the camera with the spectated surface. For this axis it is the largest and its value is around σ_z_ = 10 mm. The standard deviations of localization errors in x and y axes were set to 0.02 mm. The localization error was set equally for both input point clouds.

The covariance matrices and the cross-covariance matrix were calculated as presented in “Anisotropic ICP” with use of equations [9–13]. The principal axes matrices V were found for each point of the source and target cloud. In case of time-of-flight localization error, the principal axes were the z axis that connects the origin of camera with i-th point of the cloud and the x and y axes are perpendicular to the z axis and to each other in succeeding iterations.

As mentioned, the source cloud covariance matrix has to be recalculated after each iteration of algorithm because of updated positions of source cloud points [12]. Due to that, the cross-covariance matrix has must also be updated [11]. Once the weighting matrix has been found for an iteration [13], it can be used to modify the corresponding points distances [15].

#### Landmark’s weighting modification

Using [2] algorithm as a base method, calculation of the transformation matrix is presented next. The landmark term was rejected Eq. (5) in favor of placing the landmark correspondences in the distance term Eq. (3). Now, only the pairs of points with the closest distance that are not landmark points needed to be found. The D matrix remains unchanged, as it would in the case of isotropic non-rigid ICP algorithm. The U matrix has the landmark points’ coordinates set constant in each iteration, while the rest of point coordinates change according to the distance between point pairs. The global cost function for our method lacks the separate landmark term:

15 $$E\left( X \right) = \left\| {\left[ {\begin{array}{*{20}c} {\alpha \left( {M \otimes G} \right)} \\ {WD} \\ \end{array} } \right]X - \left[ {\begin{array}{*{20}c} 0 \\ {WU} \\ \end{array} } \right]} \right\|^{2} = \left\| {AX - B} \right\|^{2}$$

Another modification was that instead of leaving the weighting matrix W as the identity matrix, we set the weights of the points which were landmark points placed in matrix D to value ‘4’. For the rest of the points, weights were set to value ‘0.25’. This change increased the contribution of landmark points in the algorithm.

### Test data sets

#### Artificial data

The artificial data used for testing were two square, flat meshes containing 625 points each, created in Matlab environment [12]. These surfaces were generated using built in Matlab “Meshgrid” function [13]. The boundaries of the meshes were initially equal and are presented as follows: x: (− 125) mm–125 mm, y: (− 125) mm–125 mm. The z coordinates of source and target surface points were 1100 mm and 1200 mm, respectively. The surfaces have been subjected to time-of-flight localization error, the same as in the case of real data, which is: σ_z_ = 10 mm, σ_x_, σ_y_ = 0.02 mm. The purpose of this was only to visually present how finding the corresponding points is affected using the approach presented by [3].

To show what effect had the modified weighting of the landmark points on the outcome of the algorithm, the surfaces without noise have been drawn from each other creating sort of a step pattern. The alpha parameter starts at a value of 100 and decreases every 20 iterations with rate of 0.5*alpha. The maximal iteration count for testing on artificial data was 100 iterations.

It is crucial to mention that in case of artificial data, there were known correspondences of cloud points. This means that the points of one cloud correspond by their number to the points of the other cloud e.g. the first point of source cloud corresponds to the first point of target cloud, both being a part of a regular and square mesh.

#### Real clinical data

**Fig. 1:**
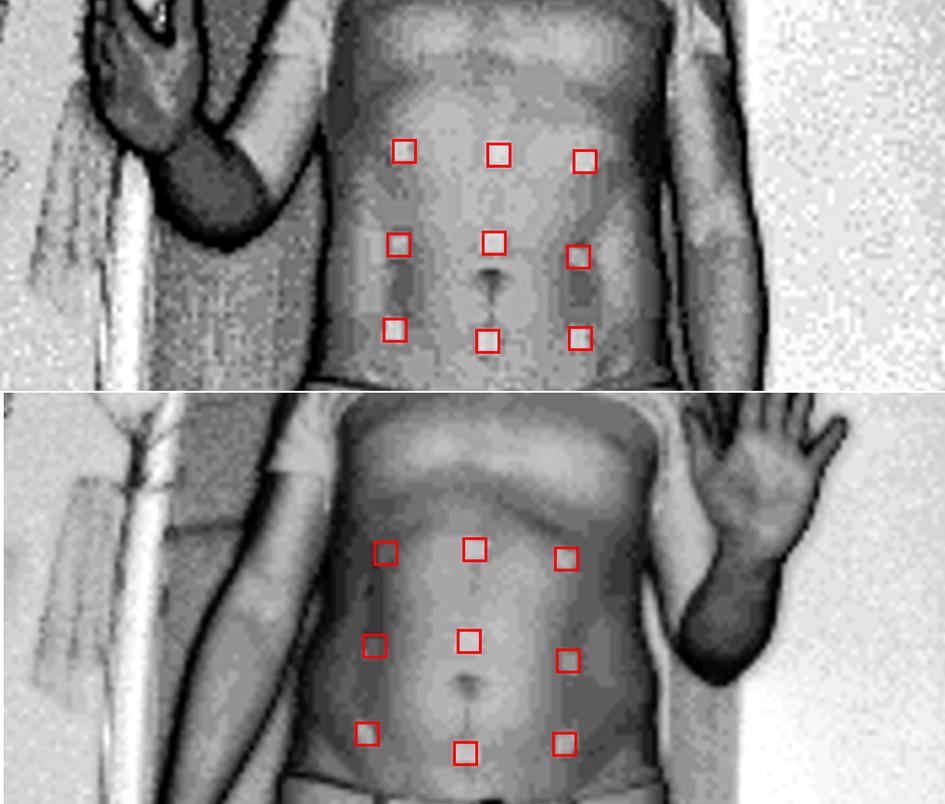
Example of real clinical data photographic image from ToF camera. Abdominal surface in moment of maximal exhalation (up) and maximal inhalation (down) phases with markers attached. Markers shown in red. Each corner of markers is considered a landmark point (marked as consecutive numbers in blue)

For measuring the suitability of our approach for human based scenarios, the algorithm was tested by registering point clouds containing data of abdominal skin surfaces during inhale and exhale phases of breathing (Fig. 1) recorded by ToF camera (depth image). This data set was chosen for testing due to our work extending modifications and results presented in [11].

The mentioned article explains usage of non-rigid Iterative Closest Point for tracking patients abdominal surfaces. This approach presents a solution for a hard case of surface registration problem. The abdominal skin changes from convex to concave during breathing phases which under geometric distance constraint, in worst case, may lead to attraction of outlying points in source cloud to closest points in the center of target cloud. No extreme folding of point surfaces should be present in this type of deformation tracking, yet the greatest change in amplitude is present for central points in cloud. The surfaces were acquired with time-of-flight camera Swiss Ranger SR4000, which has an absolute accuracy of about 1 cm. The markers used as landmark points in our algorithm were placed on the abdominal skin before the acquisition so that they were imaged with the designated object. The markers used were square-shaped and had a size of 15 mm. The frequency of the camera was set to 30 MHz, which allowed for acquisition of within a 5 m radius. The patient was at the distance of from 1 to 1.5 meters from the camera.

Our approach was tested on 10 cases of abdominal skin surface pairs each containing 9 markers distributed in a 3 × 3 mesh. From every marker, we extracted 4 landmark points which gave us 36 landmark points overall. Markers placed on patients abdominal skin were found using a tracking algorithm basing on maximization of normalized cross correlation value [14]. Such algorithm is used for searching and locating patterns present in 1D and 2D images. The maximal iteration count for testing on real data was 200 iterations.

### Selected measures of registration quality

For measuring how well the algorithm was able to register surfaces containing real data, we used three measures:

- Mean distance of the surfaces in millimeters (M1)—is measured as distance from source point clouds and their closest correspondents in target cloud (preferred minimum).
- Landmark distance in numbers of units (M2)—is the localization error of landmarks measured as sum of columns and rows by which source cloud landmark has been misaligned compared to its corresponding landmark, during registration (preferred minimum). This measure was also present in [11], named as “quality of correspondences: average correspondence assignment errors for the points nearest the markers” used for evaluation of registration quality.
- Percentage of target cloud points which had only one correspondent (M3)—the target cloud points may have multiple correspondents in source cloud, which is not desired. Great amount of points that have only one correspondences indicate that the clouds have been registered evenly/uniformly (preferred maximum).

Verifying the matching of landmark points and measuring their final distance is important because the landmark points, carry real correspondence information of the points which is set arbitrarily before the acquisition. For making this measure credible, only 9 points out of the landmarks set to play the role of landmarks were used for registration.

The remaining 27 landmark points were used as measurement points for the landmarks registration accuracy. For the 9 landmarks, the first point from each of the markers groups was chosen.

## Results

In this section we present results of testing our modification of ICP algorithm on artificial and real clinical data.

### Artificial data

For analyzing improvements of each modification of non-rigid ICP algorithm separately, we used correspondence maps, which present counts of source cloud correspondents attracted by target cloud points in color scale. The points seen on the map are the target cloud points viewed from above.

#### Anisotropic correspondence finding

**Fig. 2:**
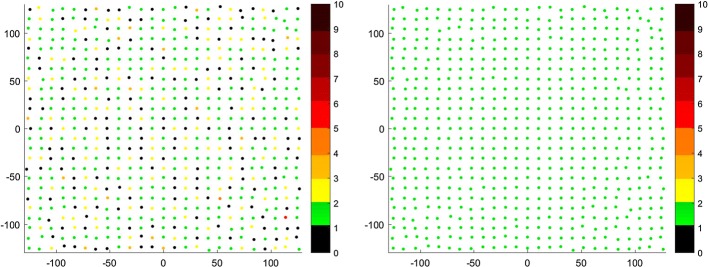
Correspondence maps for isotropic (left) and anisotropic (right) ICP. The axes present the size of the registered target point cloud. The number of correspondence for every point of target point cloud is presented in color value. The preferred value is green (one correspondence). The black value means that the point attract no correspondence, other values indicate more correspondence

First modification of non-rigid algorithm was using anisotropic approach of [3] for seeking truthful correspondences which for the isotropic variant may not be recognizable due to influence of point localization error. Below (Fig. 2), is presented the comparison of non-rigid ICP algorithm with isotropic and anisotropic alternations for correspondence finding, based on Euclidean distance of point pairs:

As we can notice, using anisotropic localization error for finding corresponding point pairs into consideration shows that every point of target cloud has single corresponding point of source cloud, while isotropic localization error has more points that have no or up to 3 corresponding points matched.

#### Increasing role of landmarks in transformation calculation

**Fig. 3:**
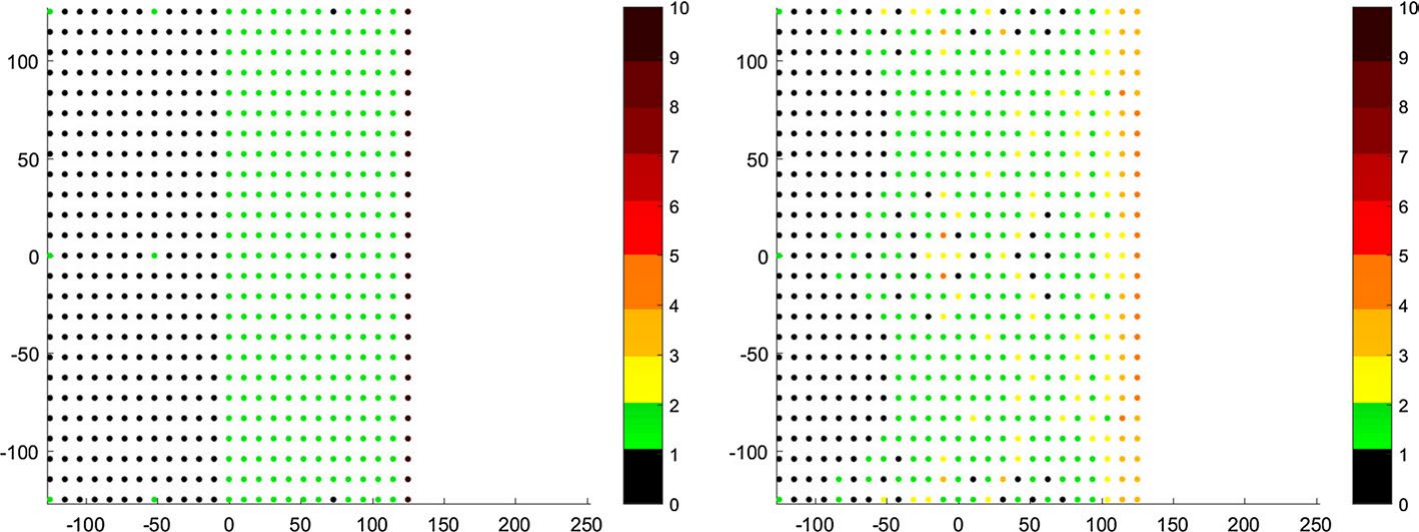
Correspondence maps for isotropic (left) and it’s modification with enhanced landmark weighting (right) ICP. The axes present the size of the registered target point cloud. The number of correspondence for every point of target point cloud is presented in color value. The preferred value is green (one correspondence). The black value means that the point attract no correspondence, other values indicate more correspondence

Another alternative is to remove the minimization of landmark term and make landmark points in the distance minimization term arbitrary. Below (Fig. 3), correspondence maps of non-rigid isotropic ICP and its modification with enhanced landmark weighting are shown. As mentioned in “Test data sets” section, the two surfaces were initially positioned remotely and spread from each other so that they formed a step-like formation. In the isotropic version of the algorithm only half of the target cloud points attracted singular or even up to ten source cloud points. In the isotropic approach with landmarks in distance minimization term and their enhanced weighting, the landmark points were able to draw some neighboring points spreading the source cloud more over the target cloud. The counts of corresponding points for each target cloud point also dropped. In first case there were points that had 10 correspondents. In the second case, points had a maximum of 5 correspondents.

### Real clinical data

As tests on artificial data showed, our approach of the anisotropic ICP algorithm allowed for an improvement of measures M2 and M3, while measure M1 was at a satisfying level of 10E−08. Table 1 describing achieved outcome of testing on real data.

**Table 1:** Quantitative evaluation of non-rigid isotropic and anisotropic ICP for real data

	Initial distance [mm]	Isotropic			Anisotropic			Anisotropic with landmark weighting		
		M1 [mm]	M2 [unit]	M3 [%]	M1 [mm]	M2 [unit]	M3 [%]	M1 [mm]	M2 [unit]	M3 [%]
C1	17.4	3.4E−08	2.3	36.1	4.68E−08	2.78	95.88	4.59E−08	1.93	84.76
C2	8.0	1.3E−08	2.8	59.1	1.43E−08	3.48	96.44	1.49E−08	1.26	78.06
C3	7.0	4.1E−09	5.7	67.4	4.34E−09	5.74	95.83	3.79E−09	4.37	78.13
C4	11.7	2.4E−08	2.1	52.1	2.59E−08	1.18	93.28	2.18E−08	1.04	84.39
C5	8.0	1.7E−08	2.9	59.0	1.9E−08	2.89	94.5	1.92E−08	1.89	78.00
C6	7.6	1.5E−08	3.7	67.4	1.39E−08	3.11	96.44	1.29E−08	1.89	83.20
C7	5.9	1.2E−08	3.3	78.4	1.14E−08	3.30	95.91	1.14E−08	3.04	79.32
C8	5.8	1.4E−08	1.9	78.6	1.5E−08	2.48	98.86	1.42E−08	*2.52*	96.59
C9	6.7	7.4E−09	5.9	76.08	7.33E−09	6.22	96.17	8.3E−10	3.89	*73.92*
Median		1.4E−08	2.9	67.36	1.43E−08	3.11	95.91	1.42E−08	1.93	79.32

The case were M2 and M3 values for Anisotropic with landmark weighting are worse than the corresponding values for the other versions of the tested method have been marked as italics

We grouped the results by ICP algorithm method, each having three measures of registration quality M1, M2 and M3 (2.6) in columns. Rows represent various point cloud pairs used for testing. Additional, initial distance of cloud pairs and the median value of each quality measure for each method has been presented. The left part presents results for non-rigid isotropic ICP algorithm, the middle one shows results for non-rigid anisotropic ICP variant and right one shows the outcome of our non-rigid anisotropic with enhanced landmark weighting approach.

There was no improvement in measures M2 and M3 only in point cloud pairs C8 and C9, respectively. In other cases, testing showed a major advantage over the plain isotropic approach. Measure M1 is at the same level for all variants. The measure M2 is almost equal for both the isotropic and anisotropic approach (except for cloud pairs C7 and C4 where there is a difference). Measure M3 was the best for the non-rigid anisotropic alternative. This is due to using [3] modification to find corresponding points in surfaces to be registered. This variant stand-alone shows about 95% of single correspondence finding.

Unfortunately, combining it with our idea of placing landmark points in distance term minimization equation as arbitrary correspondences and setting higher weights for these pair lowered the amount of single correspondences. In summary, this approach is a compromise between increasing landmarks attraction force of other points and having the most single correspondences between clouds of all.

**Fig. 4:**
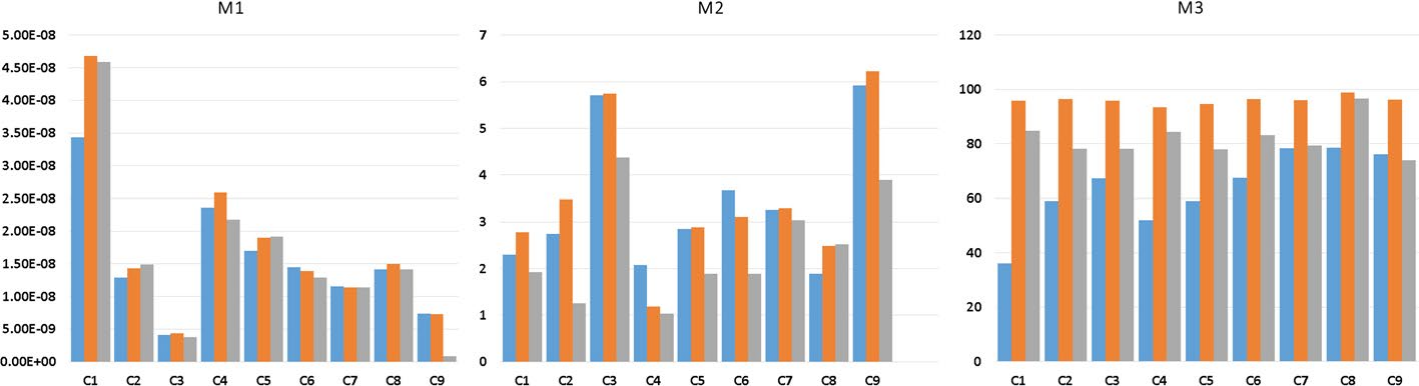
Graphical evaluation of non-rigid isotropic (blue), anisotropic (orange) and anisotropic with enhanced landmark weighting (gray) ICP for real data of the introduced measures of registration quality grouped by point cloud pairs number

Next (Fig. 4), we present figures showing measured outcome values but grouped by point cloud pairs for better visualization of improvements:

As mentioned, measure M1 is at similar level for all variations of algorithm, measures M2 and M3 have improved for almost all approaches of ICP algorithm.

**Fig. 5:**
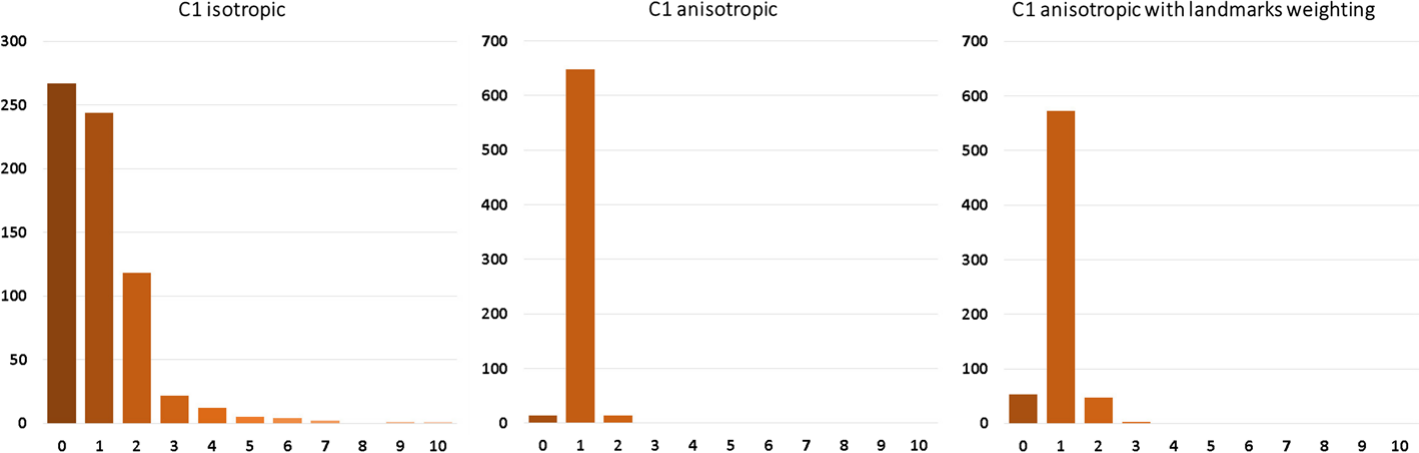
Correspondence histogram of non-rigid isotropic (left), anisotropic (middle) and anisotropic with enhanced landmark weighting (right) ICP for the greatest initial distance—C1 grouped by number of correspondence for point of target point cloud

**Fig. 6:**
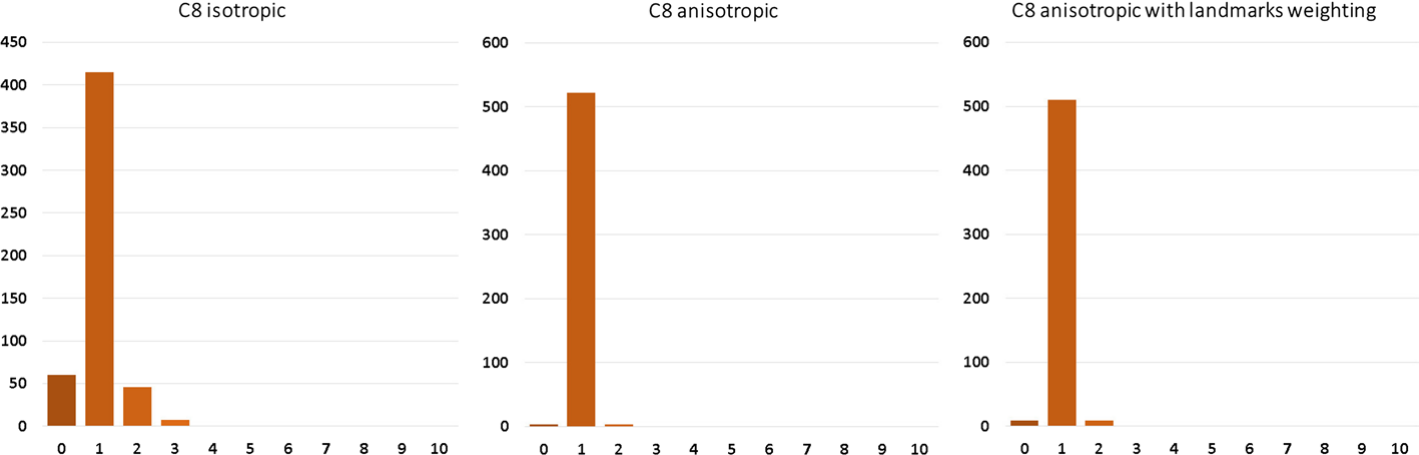
Correspondence histogram of non-rigid isotropic (left), anisotropic (middle) and anisotropic with enhanced landmark weighting (right) ICP for the least initial distance—C8 grouped by number of correspondence for point of target point cloud

**Fig. 7:**
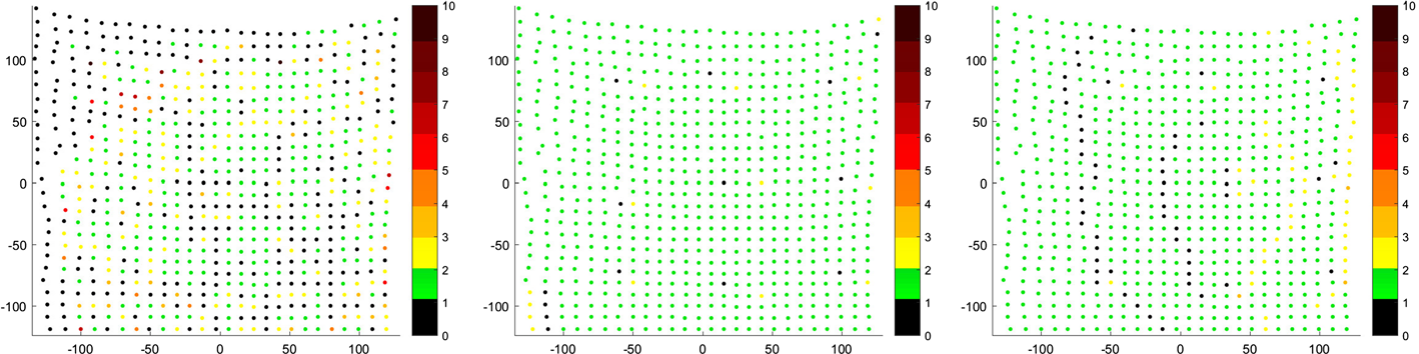
Correspondence map of non-rigid isotropic (left), anisotropic (middle) and anisotropic with enhanced landmark weighting (right) ICP for the greatest initial distance—C1. The axes present the size of the registered target point cloud. The number of correspondence for every point of target point cloud is presented in color value. The preferred value is green (one correspondence). The black value means that the point attract no correspondence, other values indicate more correspondence

**Fig. 8:**
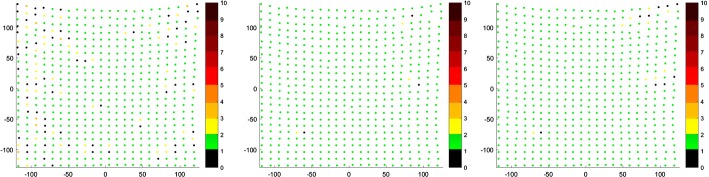
Correspondence map of non-rigid isotropic (left), anisotropic (middle) and anisotropic with enhanced landmark weighting (right) ICP for the least initial distance—C8. The axes present the size of the registered target point cloud. The number of correspondence for every point of target point cloud is presented in color value. The preferred value is green (one correspondence). The black value means that the point attract no correspondence, other values indicate more correspondence

Next, two pairs of point clouds were chosen for registration quality analysis. The first one was the one with the greatest initial distance—C1, and the other was the one with the least initial distance—C8. First, correspondence histograms are presented, which give information about how many points of target surface had certain counts of correspondents in the source surface (Figs. 5, 6 respectively):

Analyzing these histograms, we look for the most amount of single target cloud points correspondents in the source cloud. That tells, to some extent, if the point clouds have been registered uniformly or if there were points that attracted more than one source cloud point. These histograms show that the non-rigid anisotropic version of ICP algorithm achieved the most single correspondences. Just after that is approach of non-rigid ICP with strong landmark weighting. The worst results came out for the non-rigid isotropic ICP variant. As an addition to correspondence histograms the correspondence maps are presented (Figs. 7, 8 respectively):

These display which of the target cloud points had certain counts of corresponding source cloud points using color scale. Isotropic ICP has the most non-single correspondences. The anisotropic version has the best results of single correspondences with slightly worse single correspondence counts for our approach compared to non-rigid anisotropic ICP.

**Fig. 9:**
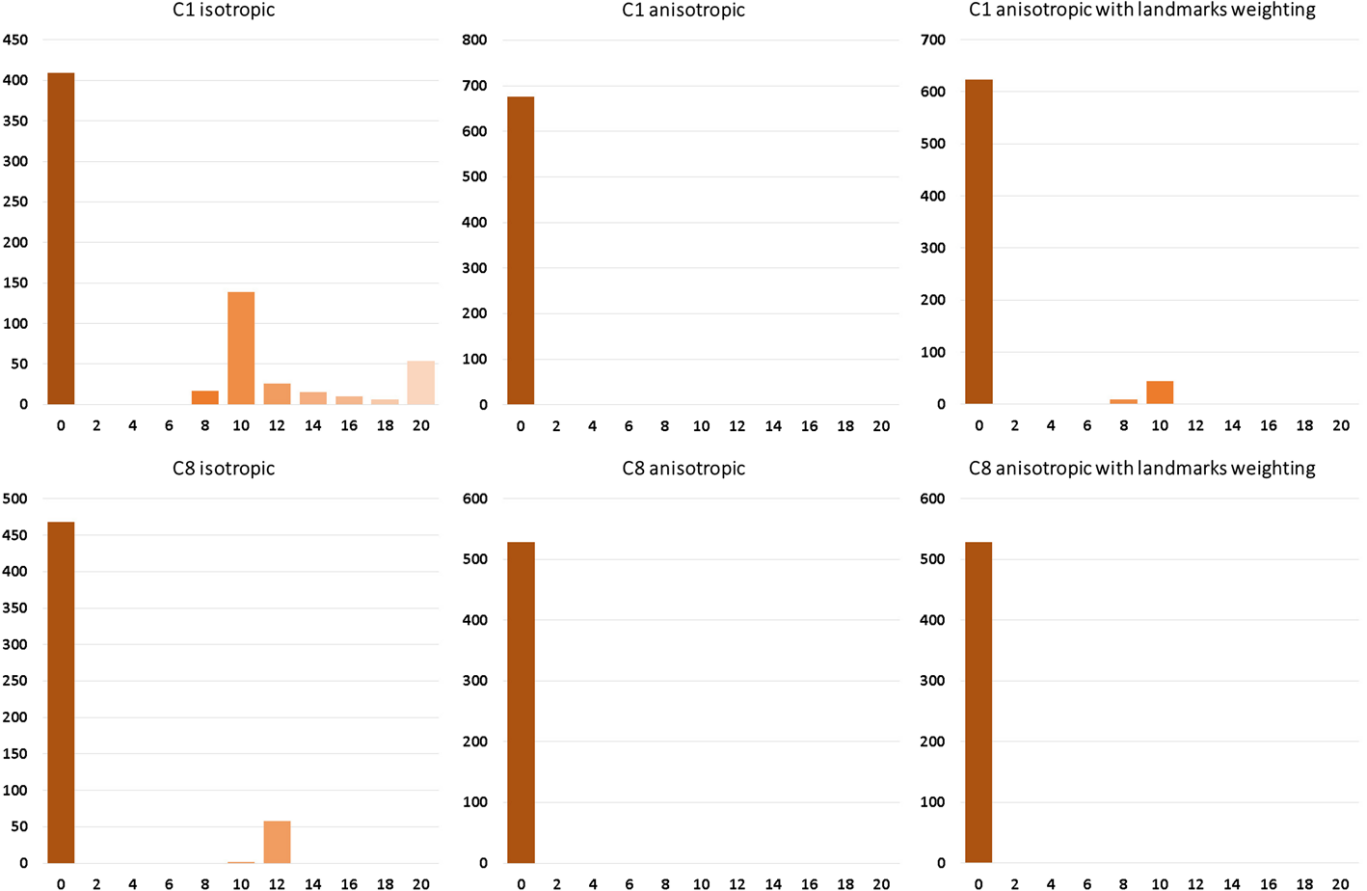
Distance map histograms of non-rigid isotropic (left), anisotropic (middle) and anisotropic with enhanced landmark weighting (right) ICP for the greatest initial distance—C1 (up) and for the least initial distance—C8 (down) grouped by the distance between corresponding points in source and target point clouds

**Fig. 10:**
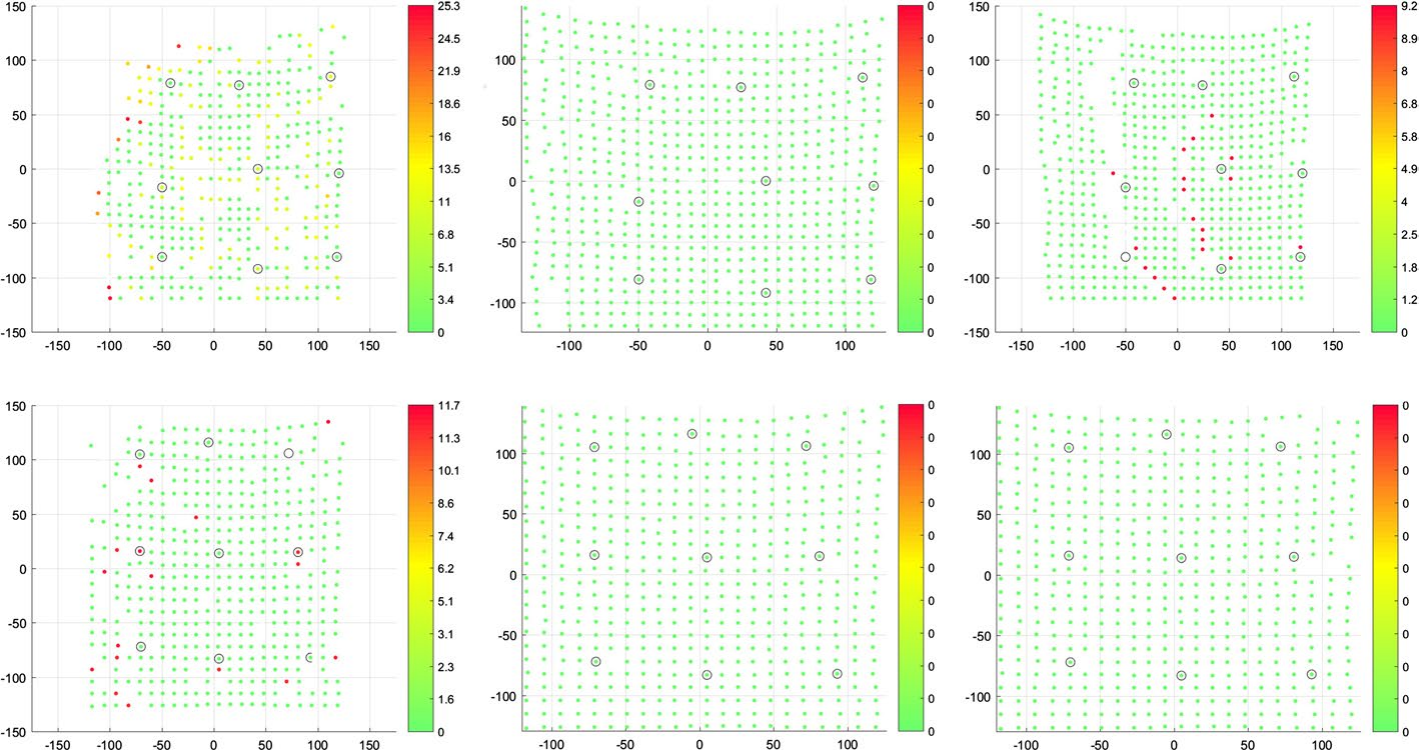
Distance maps of non-rigid isotropic (left), anisotropic (middle) and anisotropic with enhanced landmark weighting (right) ICP for the greatest initial distance—C1 (top) and for the least initial distance—C8 (bottom). The axes present the size of the registered target point cloud. The preferred value is green (distance between corresponding points in source and target points cloud equals zero)

Another histogram allowing for analysis of registration quality are the distance histograms (Fig. 9). A value of zero indicates that certain points have distance of less than 1 mm to its correspondent. Mentioned histograms are presented below:

The anisotropic version of ICP had all the point distances near zero. Isotropic ICP showed the worst outcome having, in the case of C1, 267 points in which the distance to their correspondent was greater than 8 mm. In the case of C8, few points had distances to their correspondents greater than 12 mm. Our approach is at a similar level of correspondent distance to the anisotropic variant of ICP. In the case of C1, there are some points with correspondent distances of over 8 and 10 mm. As a visual aid we present below the distance maps (Fig. 10).

These maps show which of the points in the target cloud had close-to-zero distance to their correspondent in source cloud. Holes, displayed in Fig. 2 as black dots, represent points with no correspondents. For such cases, we are not able to measure distance to corresponding point. Isotropic ICP showed the worst results because of the greater count of points with larger distances to their correspondents. The anisotropic variant presents zero distance between source and target clouds. The non-rigid anisotropic alternative with enhanced landmarks weighting shows slightly worse results than the plain anisotropic version, nevertheless it presents a better outcome than isotropic ICP.

## Discussion

Non-rigid isotropic and non-rigid anisotropic with landmark weighting ICP algorithms were compared using the following measures: mean surfaces distance (M1), mean landmark position error (M2), and percentage of target cloud points having one corresponding source cloud point (M3). We took the median of the resulting measures and the outcome was as presented: median mean of surfaces distance (M1) was at acceptable level of 10E−08 for both variants of ICP algorithm, median of mean landmark position errors (M2) decreased by 0.93 number of units, median of M3 measure increased by 11.96%.

Comparison of results of our algorithm modification with results from [3] showed a major decrease of mean surface distances. Unfortunately, that comparison is not straight forward because of the presented algorithm used the non-rigid transformation class. Results presented by [3] lack other quality measures than just the mean surface distance, compared to using three measures of quality of registration used in the approach presented in this article. Tests of the algorithm on artificial surfaces, where the real correspondences of points are known, showed that our approach enhanced the finding of correspondences which is important for registration algorithms based on the geometry of models.

In cloud pairs C8 and C9, there was no improvement. The mean landmark position error (M2) was greater in C8 and the percentage of target cloud points having one in source cloud (M3) was smaller in C9. Overall there is a compromise between the measures M2 and M3. In case of cloud pair C8, there was a significant improvement of measure M3 and worsening of measure M2. In case of cloud pair C9 the opposite occurred. This may be due to differences in the placement of landmarks in both the source and target cloud. In the case of cloud C8, measure M3 increased because the displacement of landmarks during breathing phases was minor, which lead to points in the source cloud not changing distances inside the mesh. Point cloud pair C9 shows a major displacement of landmarks between abdominal skin surfaces acquired during the inhalation and exhalation phase. This stretched the mesh and increased distances between points of the source cloud mesh. In turn, that created areas of different point density. It is more likely that a single point in the target cloud will attract a group of densely localized points rather than one point. Unlike the results of the previous, article [11], in which the influence of initial clouds distance on final outcome was seen, such phenomenon was not noticed in the approach presented in this article.

As stated in [2], the landmark point information was minimized in a separate term of the global cost function [3, 5]. This modification assumes that landmarks are set arbitrarily as corresponding points in the distance term of the global cost function which led to improvement of measure M2.

Modifications based on [3] were introduced in the first stage of the algorithm which is the matching of corresponding points. In future works, we are planning to include other modifications mentioned in [3] article, such as altering of the weight matrix in the second stage of algorithm, which is the minimization of the global cost function.

For comparison with article [11], we calculated mean values of Surface distances and marker errors from all cases and compared them with the outcome of modified algorithm, prepared using the same approach. Mentioned article evaluates presented ICP modification using data which is similar to what was used in testing of our approach. The surface distance is lesser for surfaces registered with proposed algorithm but this can be due to smaller value of stiffness parameter alpha. Other authors using non-rigid ICP, obtaining a distance of the surface, varying within 3 orders of magnitude: from thousandth of a millimeter RMS 0.0037 mm [5], through a fraction of a millimeter RMS 0.47 mm [15] to a few millimeters RMS 1.61 mm [8] and RMS 3.39 mm [10]. Lack of information on the adopted stiffness factor makes a direct comparison of numerical values unjustified.

In the presented approach the mean marker error is also reduced from 5.63 to 2.64. which indicated less offset between registered point clouds. In [4] authors showed RMSE of registered clouds and a measure of correspondents success which is similar to our M3 measure. For evaluation purposes, models of a cat, a horse and a human body were used. The improvement in single correspondence was observed for the anisotropic non-rigid Iterative Closest Point without landmark weights modification from 92.3% (Ge) to 95.5%.

## Conclusions

The paper presents a modified previously presented version of the non-rig Iterative Closest Point Algorithm by introducing an anisotropic noise model for the Time of Flight camera. This allowed to improve the results of the match quality measures. The results showed that the introduction of the anisotropic model of noise for the ToF camera allows for the improvement the percentage of target cloud points which had only one correspondent over 10% impartment and additional weighting of markers also improves the measure of the quality of finding real correspondents over 20% improvement. In the examined dataset, where the average initial distance between the clouds of points in the inspiratory and expiration is equal to approx. 7.5 mm, a more than 10% improvement in the quality of the correspondence improves the accuracy of matching the surface within 1 mm which is a significant value in application of minimally invasive image guided interventions.

In the future, the proposed method can be developed with the use of other noise models and verified in other applications.
